# The relationship between affective symptoms and hypertension—role of the labelling effect: the 1946 British birth cohort

**DOI:** 10.1136/openhrt-2015-000341

**Published:** 2016-01-12

**Authors:** Valérie Tikhonoff, Rebecca Hardy, John Deanfield, Peter Friberg, Graciela Muniz, Diana Kuh, Carmine M Pariante, Matthew Hotopf, Marcus Richards

**Affiliations:** 1 MRC Unit for Lifelong Health, Ageing at UCL, London, UK; 2 Department of Medicine, University of Padova, Padova, Italy; 3 National Centre for Cardiovascular Prevention and Outcomes, Institute of Cardiovascular Science, University College London, London, UK; 4 Department of Clinical Physiology, Sahlgrenska University Hospital, Gothenburg University, Gothenburg, Sweden; 5 Department of Psychological Medicine, King's College London Institute of Psychiatry, London, UK

## Abstract

**Objectives:**

To investigate the association between repeated measures of affective symptoms collected over 2 decades and hypertension (clinically ascertained or self-report); to test whether, among people with hypertension, affective symptoms are associated with awareness of hypertension, and to evaluate the longitudinal effects of the label of hypertension on affective symptoms.

**Methods:**

Multivariable logistic regression, accounting for confounders and mediators, were used to test the aforementioned associations in 1683 participants from a national British cohort.

**Results:**

Weak evidence of a cumulative impact of affective symptoms across adulthood on self-reported hypertension at age 60–64 years was observed (OR 1.40 (95% CI 1.10 to 1.78) and 1.19 (0.79 to 1.80) for symptoms at 1–2 time points and at 3–4 time points vs no symptoms, respectively). Study members with affective symptoms in recent times were more likely to have self-reported hypertension at age 60–64 years than those without symptoms (OR 1.47 (1.10 to 1.96)). Similar results were observed for awareness of hypertension (OR 2.00 (1.30 to 3.06)). Conversely, no associations were found with clinically ascertained hypertension. The act of labelling someone as hypertensive at age 53 years was associated with affective symptoms at age 60–64 years, independently of antihypertensive treatment and affective symptoms at the time of the diagnosis (OR 2.40 (1.32 to 4.36)).

**Conclusions:**

Our findings suggest that elevated risk of hypertension in participants with affective symptoms might be explained by awareness of hypertension and by exposure to medical attention, though not by a direct effect of affective symptoms on blood pressure. Conversely, long-term psychological consequences of the label of hypertension are observed.

**Key questions**

**What is already known about this subject?**

- The relationship between symptoms of anxiety and depression and hypertension (HT) has been studied for nearly a century and cross-sectional and longitudinal studies report conflicting results. It is unclear whether patients with affective symptoms are more or less likely to be diagnosed and treated for HT. Some authors have observed a tendency among clinicians to underdiagnose HT in those with affective symptoms (diagnostic overshadowing).
- Cross-sectional studies have demonstrated that the act of labelling someone as hypertensive may result in an increase in psychological distress by adopting a sick role, independently of blood pressure values or antihypertensive treatment status.

**What does this study add?**

- When considering the association between affective symptoms and HT, potential misclassification due to self-report should be considered.
- This study showed that patients with affective symptoms are more often diagnosed with HT due to their increased access to healthcare.
- Awareness of HT is associated with affective symptoms 10 years later after adjusting for symptoms up until diagnosis.

**How might this impact on clinical practice?**

- Our findings suggest that long-term negative psychological effects, as a consequence of the label of HT, exist. Therefore, clinicians might also consider appropriate positive health messages in patients with hypertension.

## Introduction

The relationship between affective symptoms and hypertension (HT), one of the most important conventional risk factors for cardiovascular disease (CVD), has been studied for nearly a century.^1^ Cross-sectional and longitudinal studies report conflicting results: some studies found positive associations between mental distress and HT,^2–6^ whereas others found null or even inverse associations.^7–11^

Affective symptoms may be risk factors for HT through the mediation of classic risk factors (smoking, alcohol use and physical inactivity)^12^ or due to antidepressant treatment.^9^ Alternatively, HT may be a risk factor for affective symptoms, as some studies have demonstrated that the act of labelling someone as hypertensive may result in an increase in psychological distress by adopting a sick role, independently of blood pressure (BP) values or antihypertensive treatment status.^13–15^ There are also contrasting views on whether those with affective symptoms are more or less likely to be diagnosed and treated for HT. Some authors have observed a tendency for clinicians to underdiagnose HT in those with affective symptoms (diagnostic overshadowing)^16^; others have shown that individuals with affective symptoms typically present to medical services more frequently, potentially resulting in a rise of new HT diagnoses.^17^

The inconsistent results observed in previous studies might also be due to the different methods of assessment of HT in epidemiological studies.^18^ Self-reporting of HT is determined by respondents’ knowledge that they have been diagnosed with HT, understanding of the relevant information, ability to recall it, and willingness to report it.^19^ As a consequence, when self-reported data are the sole source of information on which HT is classified, potential misclassification may lead to bias in the associations with risk factors.^20^

The aim of the present study is to investigate the association between repeated measures of affective symptoms collected over two decades and HT in late midlife in a British community-based cohort, while accounting for a large number of confounders and mediators. We test the hypothesis that any association between affective symptoms and HT is the same irrespective of the method used to define HT (clinically ascertained or self-report). We test whether, among people with HT, affective symptoms are associated with awareness of HT. We also investigate whether awareness of HT impacts subsequent affective symptoms, while accounting for antihypertensive treatment.

## Methods

### Study members

The Medical Research Council National Survey of Health and Development (NSHD) is a socially stratified sample of 5362 singleton births that took place in 1 week of March 1946 in England, Scotland and Wales.^21^ Data collections in adult life were at 36, 43 and 53 years when research nurses visited survey members in their own homes. Between 2006 and 2010 (at 60–64 years), 2856 eligible study members were invited for an assessment at one of six clinical research facilities (CRFs) or to be visited by a research nurse at home.^22^ Of those invited, 2229 (78%) were assessed: 1690 (59.2%) attended a CRF and the remaining 539 were seen at home. Of those not assessed at the CRF or at home (n=627), 31 had died, 356 refused to participate and 240 completed only a postal questionnaire. Approval was obtained from the Greater Manchester Local Research Ethics Committee and the Scotland Research Ethics Committee. Written informed consent was obtained from all study members.

### Assessment of BP and HT

At ages 53 and 60–64 years, BP was measured twice per session by a trained nurse in the participant's home or at one of the CRFs.^11^ An average of two consecutive readings was calculated. An automated digital oscillometric sphygmomanometer (Omron HEM-705, Omron Corp, Tokyo, Japan) was used. At each assessment, study members were asked by research nurses on a clinic/home visit whether they had taken any prescribed medications or tablets for high BP in the last year. Antihypertensive medications included drugs listed in the British National Formulary (BNF) in sections 2.2 (diuretics), 2.4 (β-blockers), 2.5 (HT and heart failure) and 2.6.2 (calcium channel blockers).^23^ Clinically ascertained HT was defined according to the 2013 European Society of Hypertension (ESH) and European Society of Cardiology (ESC) guidelines for the management of arterial HT as a mean systolic BP of 140 mm Hg or higher, or as a mean diastolic BP of 90 mm Hg or higher, or current use of antihypertensive medications.^24^

At the same occasions, information on HT status was also collected on postal and clinic/home visit questionnaires. Then a further variable named self-reported HT was determined by the answer to the following question “Have you been told by a doctor that you have high BP problems in the last 10 years”. As reported in other studies,^10^ ^20^ study members who responded with “yes” to this question or declared their use of antihypertensive medications were coded as having self-reported HT.

We defined aware hypertensives as those with clinically ascertained and self-reported HT, and unaware hypertensive subjects as those with clinically ascertained but no self-reported HT. Among those with clinically ascertained and self-reported HT, based on antihypertensive treatment, we further defined aware treated hypertensives and aware untreated hypertensives.

### Assessments of affective symptoms

Details of assessments of symptoms of anxiety and depression have been described previously.^11^ Briefly, at age 36 years, a short version of the Present State Examination (PSE), a clinically validated interview administrated by trained nurses, was used; the Index of Definition (PSE-ID) provides a scale of severity ranging from 1 to 7, with a threshold for caseness of 5 or more. At age 43 years, the Psychiatric Symptom Frequency (PSF) scale, an interview-based 23-item scale, was administered with a validated threshold for potential caseness between 22 and 23. At ages 53 and 60–64 years, study members completed the 28-item self-administered General Health Questionnaire (GHQ-28), which correlates highly with the PSE.^11^

Treatment with antidepressant medications was coded at all these ages, and included any drug listed in the BNF section 4.3 (tricyclic antidepressants, monoamine-oxidase inhibitors, selective serotonin reuptake inhibitors, and other antidepressant drugs).^23^

### Covariates

Factors that could confound or mediate the main associations were identified a priori. A full description of the following covariates was extensively described elsewhere.^11^ ^22^ Data on smoking, drinking and physical activity at ages 53 and 60–64 years as well as data on educational attainment by age 26 years and socioeconomic position (SEP) at age 53 years were extracted from self-completed questionnaires, nurse interviews and diet diaries.

At age 53 and 60–64 years, body mass index (BMI) was calculated as weight in kilograms divided by height in metres squared. Heart rate was measured by the automated digital oscillometric sphygmomanometer cited above. Diabetes mellitus was a self-reported diagnosis confirmed by general practitioners, or a fasting blood glucose concentration of at least 7.0 mmol/L, or glycated haemoglobin of at least 6.5%, or use of insulin or oral antidiabetic agents. History of CVD included non-fatal myocardial infarction, acute coronary syndrome, surgical and percutaneous coronary revascularisation, angina pectoris, chronic ischaemic heart disease, stroke and heart failure.

### Statistical methods

We used χ^2^ and Student t tests to examine differences in characteristics of study members by clinically ascertained and self-reported HT status at age 60–64 years and, among hypertensives, by awareness HT status.

We created two lifetime affective caseness variables: a cumulative variable based on the number of times an individual was classified as a ‘case’ during the follow-up (no symptoms, case-level symptoms at 1–2 time points and case-level symptoms at 3–4 time points) and an age-specific variable based on the specific timing an individual was classified as a ‘case’ during the follow-up (no symptoms, case-level symptoms only in the past (age 36 and/or 43 and/or 53 years) and case-level symptoms in recent times). We first test the hypothesis that any association between affective symptoms and HT is the same irrespective of the method used to define HT. Multivariable logistic regression models were used to compute ORs with 95% CIs of being a clinically ascertained HT case, and separately a self-reported HT case at age 60–64 years by lifetime affective caseness variables (cumulative and age-specific), initially unadjusted (model 1) and then adjusted for the confounders, gender, BMI at age 60–64 years, educational attainment, SEP, and history of CVD and diabetes mellitus at age 60–64 years (model 2). Further adjustments were then made for the potential mediators’ heart rate, smoking status, drinking status, physical activity and antidepressant treatment at age 60–64 years (model 3).

Using multivariate logistic regressions adjusted for the covariates aforementioned, we tested whether, among people with HT, affective symptoms are associated with awareness of HT (aware vs unaware hypertensives) at age 60–64 years. We additionally adjusted for duration of clinically ascertained HT in years (model 4).

Finally, to investigate possible causal direction (awareness of HT preceding development of affective symptoms), we tested the association between awareness of HT at age 53 years and affective symptoms at age 60–64 years, with adjustments for affective symptoms at age 53 years to account for existing symptoms, gender and other covariates at age 53 years (namely BMI, SEP, history of CVD and diabetes mellitus, heart rate, smoking and drinking status, physical activity and antihypertensive treatment). This analysis is based on 1619 study members with available data on BP and other covariates at age 53 years and affective symptoms at age 60–64 years. To further investigate the potential impact of the antihypertensive treatment, we created at age 53 years an awareness HT status by an antihypertensive treatment variable: unaware hypertensives (reference), aware treated hypertensives and aware untreated hypertensives. We then tested the association between this variable and affective symptoms at age 60–64 years with adjustments for the same covariates as above.

Analyses were repeated (1) in study members with incomplete data on affective symptoms across adulthood and complete case information on all variables included up to model 4 (sex, BMI at age 60–64 years, education, SEP at age 53 years, history of CVD and diabetes mellitus at age 60–64 years) to maximise the sample size (n=1905); (2) considering a greater level of severity of HT (systolic BP of 160 mm Hg or higher, or as diastolic BP of 95 mm Hg or higher) and (3) considering awareness of HT in controlled and uncontrolled treated hypertensives.

The analyses presented, unless otherwise specified, are based on the sample with complete data on affective symptoms at all four ages, all covariates and at least one of the outcome measures (n=1683). Analyses were conducted using SAS software, V.9.3 (SAS Institute, Cary, North Carolina, USA).

## Results

### Characteristics of participants

**Figure 1:**
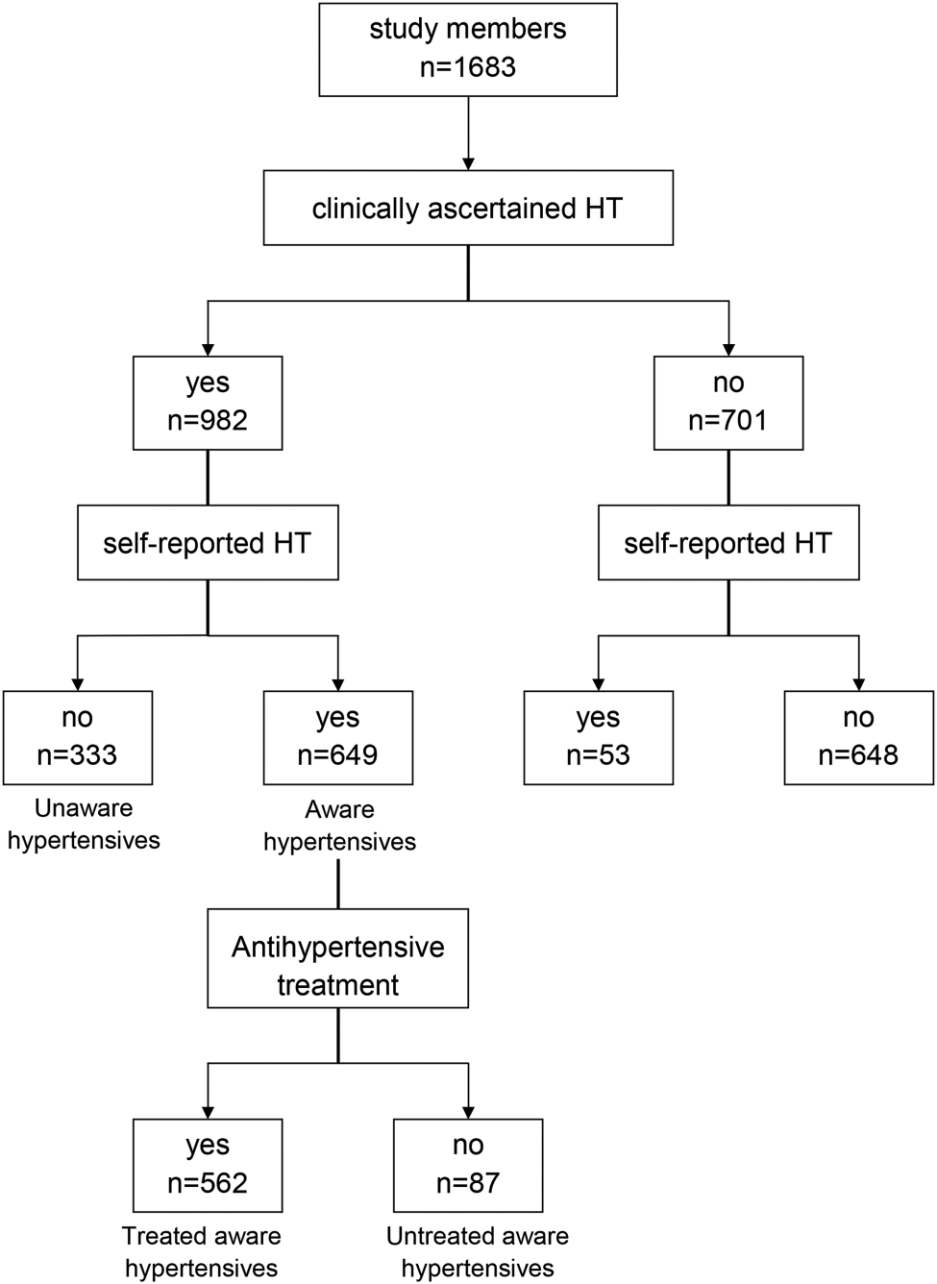
Flow chart of the 1683 study members at age 60–64 years by hypertension (HT) status. Clinically ascertained HT is based on the European Society of Hypertension (ESH)/European Society of Cardiology (ESC) 2013 guidelines (normotensives are patients with systolic blood pressure (SBP) <140 and diastolic blood pressure (DBP) <90 mm Hg and no medication use). Self-reported HT is based on self-completed questionnaires (normotensives are patients with negative reply on the question about HT knowledge and negative reply on question on medication use).

The flow chart of the 1683 study members at age 60–64 years by HT status (clinically ascertained and self-reported) is illustrated in figure 1. The prevalence of clinically ascertained and self-reported HT was 58.3% and 41.7%, respectively. Two-thirds of the study members with HT (66.1%) were aware of HT (ie, with both clinically ascertained and self-reported HT) and one-third (33.9%) were unaware of it (ie, with HT but no self-reported HT). Only 7.6% self-reported HT while they had a normal BP value and were not on antihypertensive treatment. The percentage of study members with self-reported HT based only on the questionnaire on antihypertensive medication was 9.5% (n=67), 53 of whom were also assigned to the clinically ascertained HT group. Among the 562 treated hypertensives, 51.6% had normalised BP (systolic BP<140 mm Hg and diastolic BP<90 mm Hg). Among the 420 untreated hypertensives, only 20.7% self-reported that they had high BP (aware untreated hypertensives).

Table 1 summarises the general characteristics of the 1683 study members (52.4% women) at age 60–64 years by clinically ascertained and self-reported HT status.

**Table 1:** Characteristics of the study participants at age 60–64 years by clinically ascertained and self-reported HT (n=1683)

		Clinically ascertained HT*			Self-reported HT†		
	All participants (n=1683)	Normotensive (n=701)	Hypertensive (n=982)	p Value	Normotensive (n=981)	Hypertensive (n=702)	p Value
Characteristics							
Clinically ascertained HT, n (%)*	982 (58.3)	–	–	–	333 (33.9)	649 (92.4)	<0.0001
Self-reported HT, n (%)†	702 (41.7)	53 (7.6)	649 (66.1)	<0.0001	–	–	–
Gender (women), n %	883 (52.4)	417 (59.5)	466 (47.5)	<0.0001	527 (53.7)	356 (50.7)	0.22
Clinical features							
SBP (mm Hg)‡	136.2±18.0	124.1±0.6	144.9±0.5	<0.0001	133.0±0.7	140.6±0.6	<0.0001
Diastolic blood pressure (mm Hg)‡	77.8±9.8	72.9±0.3	81.9±0.3	<0.0001	76.6±0.3	79.5±0.4	<0.0001
Heart rate (bpm)	68.6±11.0	67.8±0.4	69.2±0.3	0.009	68.4±0.4	68.9±0.4	0.42
Body mass index (kg/m^2^)§	28.0±4.9	26.4±0.2	29.1±0.2	<0.0001	27.0±0.1	29.4±0.2	<0.0001
Questionnaire data							
Educational achievement at age 26 years (higher level), n (%)	682 (40.5)	309 (44.1)	373 (37.9)	0.01	412 (42.0)	270 (38.5)	0.14
SEP at age 53 years (non-manual skill), n (%)	1134 (67.4)	487 (69.5)	647 (65.9)	0.12	687 (70.0)	447 (63.7)	0.006
Smokers (current), n (%)	190 (11.3)	80 (11.4)	110 (11.2)	0.89	106 (10.8)	84 (12.0)	0.46
Drinkers (≥5 g/day), n (%)	1067 (63.4)	537 (76.6)	707 (72.0)	0.03	747 (76.2)	497 (70.8)	0.014
Leisure time physical activity (inactive), n (%)	1059 (63.0)	414 (59.1)	645 (65.7)	0.006	590 (60.1)	469 (66.8)	0.005
Antihypertensive treatment, n (%)	562 (33.4)	0 (0.0)	562 (57.2)	–	0 (0.0)	562 (80.1)	–
Diabetes mellitus, n (%)	164 (9.7)	29 (4.1)	135 (13.7)	<0.0001	47 (4.8)	117 (16.7)	<0.0001
History of CVD, n (%)	212 (12.6)	42 (6.0)	170 (17.3)	<0.0001	66 (6.7)	146 (20.8)	<0.0001
Affective symptoms, n (%)¶	298 (17.7)	119 (17.0)	179 (18.2)	0.51	149 (15.2)	149 (21.2)	0.001
Antidepressant treatment, n (%)	130 (7.7)	58 (8.3)	72 (7.3)	0.47	69 (7.0)	61 (8.7)	0.20

Values are arithmetic means±SE or number of participants (%).

*Clinically ascertained HT=definition based on the ESH/ESC 2013 guidelines (normotensives are patients with SBP<140 and DBP<90 mm Hg and no medication use).

†Self-reported HT=definition based on the questionnaire (normotensives are patients with a negative reply on the question about HT knowledge and a negative reply on the question on medication use).

‡Average of two blood pressure readings.

§The body mass index is weight in kilograms divided by the square of the height in metres.

¶Total GHQ-28 score ≥5.

CVD, cardiovascular disease; DBP, diastolic blood pressure; ESC, European Society of Cardiology; ESH, European Society of Hypertension; GHQ-28, General Health Questionnaire–28-items; HT, hypertension; SBP, systolic blood pressure; SEP, socioeconomic position.

### Affective symptoms and clinically ascertained and self-reported HT at age 60–64 years

When we considered the association between the cumulative lifetime affective variable and self-reported HT (table 2), in the unadjusted analysis study members reporting affective symptoms at 1–2 time points and at 3–4 time points were more likely to have self-reported HT than those without symptoms (p value for trend 0.001). In the fully adjusted model, the association in those with affective symptoms at 3–4 time points was considerably attenuated, providing little evidence of a cumulative impact of affective symptoms across adulthood on self-reported HT at age 60–64 years (p value for deviation from linear trend 0.08).

**Table 2:** Logistic regression between clinically ascertained and self-reported HT at age 60–64 years and lifetime affective caseness variables (n=1683)

	Clinically ascertained HT vs normotension (982 vs 701)							
	*Cumulative lifetime affective caseness* (OR (95% CI))*				*Age-specific lifetime affective caseness* (OR (95% CI))*			
	No symptoms (n=1080)	Case-level symptoms at 1–2 time points (n=474)	Case-level symptoms at 3–4 time points (n=129)	p Value†	No symptoms (n=1080)	Case-level symptoms only in the past (n=305)	Case-level symptoms in recent times (n=298)	p Value†
Model 1	1.00	0.99 (0.79 to 1.23)	1.10 (0.76 to 1.59)	0.87	1.00	0.95 (0.74 to 1.23)	1.08 (0.83 to 1.40)	0.75
Model 2	1.00	0.98 (0.77 to 1.24)	0.98 (0.64 to 1.47)	0.83	1.00	0.93 (0.70 to 1.22)	1.04 (0.78 to 1.38)	0.79
Model 3	1.00	1.01 (0.79 to 1.28)	1.01 (0.66 to 1.54)	0.84	1.00	0.95 (0.72 to 1.26)	1.07 (0.80 to 1.43)	0.80
Self-reported HT vs normotension (702 vs 981)								
Model 1	1.00	1.44 (1.16 to 1.80)	1.48 (1.03 to 2.14)	0.001	1.00	1.32 (1.02 to 1.71)	1.60 (1.24 to 2.07)	0.0006
Model 2	1.00	1.41 (1.11 to 1.78)	1.22 (0.81 to 1.83)	0.004	1.00	1.27 (0.96 to 1.67)	1.48 (1.12 to 1.97)	0.01
Model 3	1.00	1.40 (1.10 to 1.78)	1.19 (0.79 to 1.80)	0.02	1.00	1.26 (0.95 to 1.67)	1.47 (1.10 to 1.96)	0.02

Model 1: unadjusted logistic regression.

Model 2: logistic regression adjusted for gender, BMI at age 60–64 years, educational attainment by age 26 years, socioeconomic position at age 53 years, history of CVD and diabetes mellitus status at age 60–64 years.

Model 3: model 2 additionally adjusted for covariates at age 60–64 years: heart rate, current smoking, alcohol consumption, physical activity and antidepressant treatment.

*Affective caseness assessed at each time point as follow: PSE-ID≥5 at age 36 years, total PSF score ≥23 at age 43 years, and total GHQ-28 score ≥5 at ages 53 and 60–64 years.

†p Value for trend.

BMI, body mass index; CVD, cardiovascular disease; GHQ-28, General Health Questionnaire–28-items; HT, hypertension; PSE-ID, Present State Examination-Index of Definition; PSF, Psychiatric Symptom Frequency.

There was evidence of an association between the age-specific lifetime affective variable and self-reported HT (table 2). Study members with affective symptoms only in the past and those with symptoms in recent times were more likely to have self-reported HT than those without symptoms, with the OR for those with symptoms in recent times being greater (p value for heterogeneity across groups 0.02). There was no evidence of associations between cumulative and age-specific lifetime affective variables and clinically ascertained HT (table 2).

Case-level affective symptoms at each of ages 36, 43, 53 and 60–64 years were not associated with odds of clinically ascertained HT at ages 60–64 years at all levels of covariate adjustment (see online supplementary table S1). In contrast, in the fully adjusted model, only those with case-level affective symptoms at 60–64 years had greater odds of self-reported HT at the same age (OR 1.38; 95% CI 1.04 to 1.81) (see online supplementary table S2).

### Affective symptoms and awareness of HT at ages 60–64 years

Aware hypertensives at ages 60–64 years (n=649) compared with those unaware (n=333) were more likely to be female, to have lower systolic BP and diastolic BP, to have higher BMI, to be physically inactive, to have a higher prevalence of diabetes mellitus, history of CVD, affective symptoms at ages 60–64 years, to be treated with antidepressants and antihypertensive drugs and to be hypertensives at age 53 years than those unaware (see online supplementary table S3).

When we considered the association between the cumulative lifetime affective variable and awareness of HT (table 3), in unadjusted analyses study members reporting affective symptoms at 1–2 time points and at 3–4 time points were more likely to be aware of HT than those without symptoms. In the fully adjusted model, the association in those with affective symptoms at 3–4 time points was considerably attenuated, providing little evidence of a cumulative impact of affective symptoms across adulthood on awareness of HT at age 60–64 years.

**Table 3:** Logistic regression between awareness of hypertension at age 60–64 years and lifetime affective caseness variables (n=982)

	Aware vs unaware hypertensives (649 vs 333)							
	*Cumulative lifetime affective caseness* (OR (95% CI))*				*Age-specific lifetime affective caseness* (OR (95% CI))*			
	No symptoms (n=629)	Case-level symptoms at 1–2 time points (n=275)	Case-level symptoms at 3–4 time points (n=78)	p Value†	No symptoms (n=629)	Case-level symptoms only in the past (n=174)	Case-level symptoms at recent time (n=179)	p Value†
Model 1	1.00	2.20 (1.60 to 3.05)	1.78 (1.05 to 3.00)	<0.0001	1.00	1.94 (1.33 to 2.82)	2.28 (1.55 to 3.55)	<0.0001
Model 2	1.00	1.97 (1.40 to 2.76)	1.45 (0.83 to 2.52)	0.003	1.00	1.72 (1.16 to 2.55)	1.93 (1.32 to 2.96)	0.0005
Model 3	1.00	1.90 (1.34 to 2.67)	1.34 (0.76 to 2.36)	0.001	1.00	1.66 (1.11 to 2.48)	1.87 (1.24 to 2.82)	0.002
Model 4	1.00	1.91 (1.34 to 2.72)	1.55 (0.86 to 2.78)	0.001	1.00	1.67 (1.10 to 2.53)	2.00 (1.30 to 3.06)	0.001

Aware hypertensives=patients with both clinically ascertained and self-reported hypertension.

Unaware hypertensives=patients with clinically ascertained hypertension but not self-reported hypertension.

Model 1: unadjusted logistic regression.

Model 2: logistic regression adjusted for gender, body mass index (BMI) at age 60–64 years, educational attainment by age 26 years, socioeconomic position at age 53 years, history of cardiovascular disease (CVD) and diabetes mellitus status at age 60–64 years.

Model 3: model 2 additionally adjusted for covariates at age 60–64 years: heart rate, current smoking, alcohol consumption, physical activity and antidepressant treatment.

Model 4: model 3 additionally adjusted for duration of clinically ascertained hypertension in years from age 36 years.

*Affective caseness assessed at each time point as follow: Present State Examination-Index of Definition (PSE-ID) ≥5 at age 36, total Psychiatric Symptom Frequency (PSF) score ≥23 at age 43, and total General Health Questionnaire–28-items (GHQ-28) score ≥5 at ages 53 and 60–64 years.

†p Value for trend.

There was evidence of an association between the age-specific lifetime affective variable and awareness of HT (table 3). In unadjusted models, study members with affective symptoms only in the past and those with symptoms in recent times were more likely to be aware of HT at age 60–64 years than those without symptoms, with the OR for those with symptoms in recent times being greater (p value for heterogeneity across groups <0.0001). These results were similar after adjustment for lifestyle behaviours and antidepressant medication at age 60–64 years (model 3). The additional adjustment for duration of clinically ascertained HT strengthened the association (model 4).

### Awareness of HT at age 53 years and affective symptoms at age 60–64 years

The prevalence of clinically ascertained HT at age 53 years was 45.7% (n=769) and 39.0% (n=300) were aware of HT (see online supplementary table S3). No differences on systolic and diastolic BP, and antidepressant treatment were observed by awareness HT status at age 53 years, while aware hypertensives were more likely to have a lower heart rate and lower educational level and adult SEP, and to be non-smokers, non-drinkers and physically active than those unaware.

When we considered the association between awareness of HT at age 53 years and affective symptoms at age 60–64 years adjusted for gender, affective symptoms and other covariates at age 53 years, aware hypertensives were more likely to have affective symptoms at age 60–64 years than those unaware (OR 1.90, 95% CI 1.24 to 2.91; p=0.002). These results were similar after adjustment for antihypertensive treatment at age 53 years (OR 2.40, 95% CI 1.32 to 4.36; p=0.004).

In unadjusted and fully adjusted models, both aware treated hypertensives and aware untreated hypertensives at age 53 years were more likely to have affective symptoms at age 60–64 years than those unaware (table 4).

**Table 4:** Logistic regression between affective symptoms at age 60–64 years and awareness of HT at age 53 years (n=755)

Case of affective symptoms at age 60–64 years vs non-case (127 vs 628)*				
	Awareness of HT at age 53 years by antihypertensive treatment status (OR (95% CI))			
Predictor	Unaware (n=460)	Aware treated (n=212)	Aware untreated (n=83)	p Value†
Model 1	1.00	2.33 (1.53 to 3.56)	2.49 (1.41 to 4.41)	<0.0001
Model 2	1.00	1.86 (1.71 to 2.97)	2.40 (1.34 to 4.29)	0.003
Model 3	1.00	1.69 (1.04 to 2.74)	2.40 (1.32 to 4.36)	0.007

Unaware hypertensives=patients with clinically ascertained but not self-reported HT.

Aware treated hypertensives=patients with antihypertensive treatment with both clinically ascertained and self-reported HT.

Aware untreated hypertensives=patients without antihypertensive treatment with both clinically ascertained and self-reported HT.

Model 1: unadjusted logistic regression.

Model 2: logistic regression adjusted for gender, BMI at age 53 years, educational attainment by age 26 years, socioeconomic position at age 53 years and the following covariates at age 53 years: history of CVD and diabetes mellitus status, heart rate and lifestyle behaviours.

Model 3: model 2 additionally adjusted for affective symptoms at age 53 years.

*Affective caseness assessed as total GHQ-28 score ≥5.

†p Value for trend.

BMI, body mass index; CVD, cardiovascular disease; GHQ-28, General Health Questionnaire–28-items; HT, hypertension.

### Sensitivity analysis

In the larger sample of 1905 study members, we confirmed the fully adjusted findings of table 2 (case-level symptoms at 1–2 time points OR 1.38, 95% CI 1.11 to 1.72 and case-level symptoms at 3–4 time points OR 1.17, 95% CI 0.79 to 1.72; p value for trend 0.02; and case-level symptoms only in the past OR 1.21, 95% CI 0.94 to 1.57 and case-level symptoms in recent times OR 1.49, 95% CI 1.14 to 1.96; p value for trend 0.01).

Analyses using the higher cut-off values for the diagnosis of HT resulted in similar but weaker findings than those from table 3 (see online supplementary table S4).

Analysis considering BP control achievement at age 53 years in patients with hypertension resulted in similar findings than those from table 4 (see online supplementary table S5).

## Discussion

In a nationally representative British sample, for the first time evidence was found for an association between awareness of HT at age 53 years and affective symptoms 10 years later after adjustment for symptoms up until diagnosis, which suggests a causal association from diagnosis to symptoms. This association was independent of antihypertensive treatment. Conversely, an association was observed between affective symptoms and self-reported HT at age 60–64 years, while no association was found with clinically ascertained HT. Affective symptoms in recent times were strongly associated with self-reported HT at age 60–64 years, while the cumulative impact of affective symptoms across adulthood was weak. The association, robust to adjustment for a wide range of potential confounders, was not explained by lifestyle behaviours or use of antidepressant medication. Finally, an association was also observed between the presence of affective symptoms in recent times and awareness of HT at age 60–64 years. Duration of clinically ascertained HT did not influence this association.

**Figure 2:**
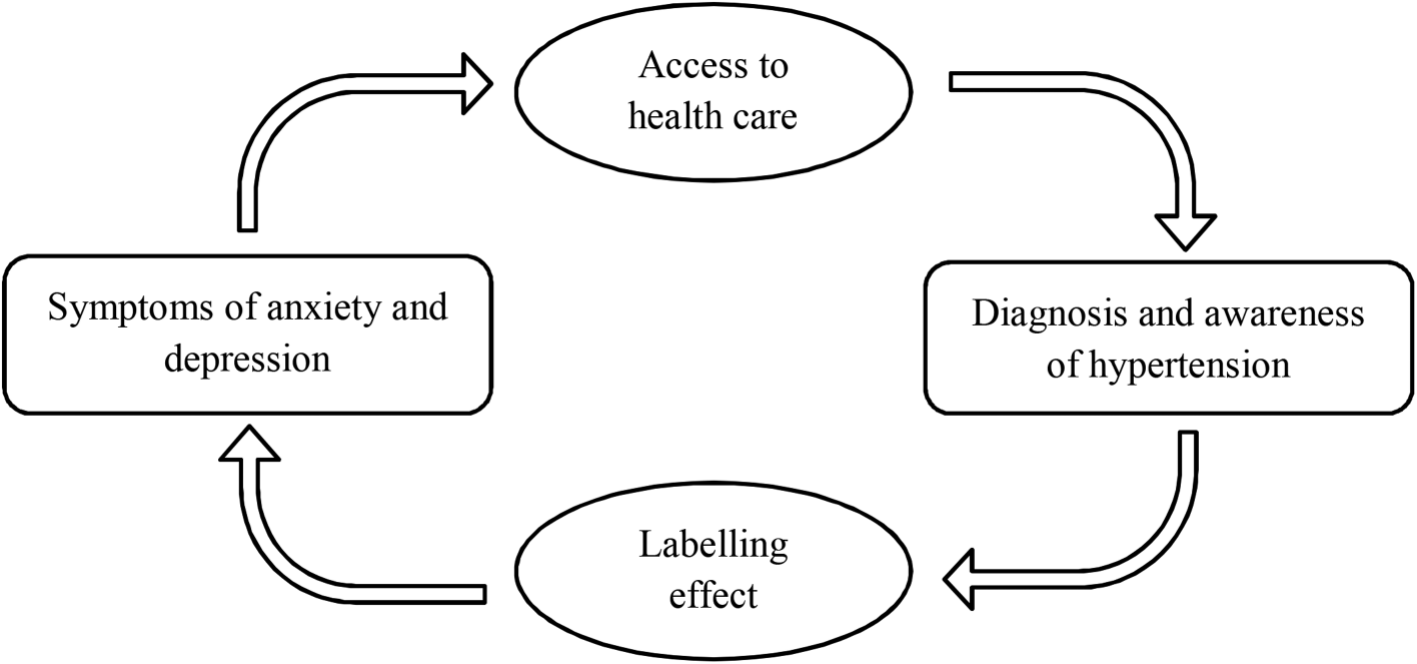
Schematic overview of the relationship between symptoms of anxiety and depression and hypertension.

As illustrated in figure 2, the association observed among people aware of their HT status may reflect the impact of having an HT diagnosis on affective symptoms, termed the labelling effect. Cross-sectional studies have demonstrated higher psychological distress and lower well-being in labelled hypertensives and mislabelled normotensives in comparison with unaware hypertensives.^13^ Being labelled as hypertensive, whether or not individuals are treated, or whether BP is within normal range (ie, mislabelled normotensives) or high, is associated with similar adverse responses.^25^ Only one prospective study reported that a greater number of newly treated hypertensives reported increased awareness of symptoms by 6-month follow-up.^26^

Our findings also suggest that the association between affective symptoms and HT seems to be explained by study members’ knowledge and understanding of HT itself rather than elevated BP. Indeed, the association between affective symptoms across adulthood and systolic BP in early old age is actually an inverse one in this cohort.^11^ Those with affective symptoms may be more likely to seek medical advice and thus be diagnosed with HT (figure 2). In this respect, Robinson^27^ found that patients who consulted a doctor and were diagnosed as hypertensive did not differ in depression scores from those diagnosed as normotensive, but both groups were more depressed than individuals who did not consult a doctor.

Finally, our results suggest that the method of ascertainment of HT is of paramount importance. Previous studies based on self-reported HT have generally found a positive association with depression;^3–4^ ^6^ ^28^ whereas studies based on clinically ascertained HT reported no association with depression.^7–9^ ^29^ In a meta-analysis that included 83 studies on BP and personality, Jorgensen *et al*^30^ found an association between high BP and negative affect when individuals were aware of their BP levels, but a reverse association when they were unaware.^30^ The few prior studies with both HT definitions available found that affective symptoms were associated with self-reported HT, but not with actual elevation in BP.^17^ ^18^

### Strengths and limitations of this study

The main strength of this study is the longitudinal collection of information on affective symptoms and on both clinically ascertained and self-reported HT across adulthood in a nationally representative cohort. This allowed us to study the relationship between lifetime affective symptoms and HT taking into account the method used to define HT, and also to investigate for the first time a possible causal direction in the association (awareness of HT preceding development of affective symptoms). The importance of repeated assessments when evaluating the effect of mental distress on HT has been emphasised, because of the fluctuation of affective symptoms over time.^5^ The increasing number of assessments provides a more precise exposure measure.

Study limitations include the use of different measures of affective caseness over time. However, each measure has its own validated threshold for case-level symptoms. The reliance on two BP measurements taken during a single physical examination instead of multiple measurements over longer time intervals may have led to biased estimates of clinically ascertained HT prevalence. The presence of white coat HT may induce an overestimation of the prevalence of clinically ascertained HT and a concomitant underestimate of the prevalence of self-reported HT.^20^ However, this should have strengthened the association with clinically ascertained HT, if anything. Different antihypertensive drug classes might have either positive or negative effects on affective symptoms and, considering antihypertensive treatment status as a whole, cannot completely take into account the impact of antihypertensive drug classes on affective status. A selection bias may have also occurred due to more frequent contact with healthcare services in study members with affective symptoms.^25^ Indeed, they may be more likely to be diagnosed with HT. This is apparently in contrast to “diagnostic overshadowing”, a process by which physical symptoms are misattributed to mental illness in a clinical setting.^16^ However, HT is often symptomless, which implies that HT may be present long before diagnosis. This suggests the possibility that aware hypertensives are a select group who may be more likely to visit a doctor. Finally, we could not exclude that a common neurological factor may lead to both a tendency to depression/anxiety and HT as autonomic deregulation.

From the 2216 study members with at least one valid outcome measure, 533 were excluded from analyses because of missing covariate data. Those excluded did not differ in frequency of self-reported HT but were more likely to have clinically ascertained HT compared with those included. In population studies, there is compelling evidence that those with affective symptoms are more prone to be non-participants. The NSHD cohort was established using a socially stratified sampling frame that ensured that it was broadly representative of the population born in England, Scotland and Wales in 1946. Since then, losses to follow-up due to death, emigration, loss of contact and permanent refusal have occurred. Despite this, at age 60–64 years, the sample remained representative in many respects of the national population born at a similar time.^22^

## Conclusions and clinical implications

The most interesting clinical implication of our study is the long-term negative psychological impact of HT labelling. A definitive study would be to assess the long-term implication of such a labelling effect on well-being and quality of life, and the potential impact on cardiovascular outcomes. Our findings might also partly explain why prior studies have produced mixed findings regarding the association of HT with common mental disorder. When considering the association between affective symptoms and HT, potential misclassification due to self-report should be considered. We also observed that patients with affective symptoms are apparently more often diagnosed with HT due to their increased access to healthcare. Further studies are required to confirm this observation and to investigate if this is peculiar to diagnosis of HT or may be extended to other major diseases, such as diabetes mellitus.
